# Comparison of the oncogenomic landscape of canine and feline hemangiosarcoma shows novel parallels with human angiosarcoma

**DOI:** 10.1242/dmm.049044

**Published:** 2021-07-23

**Authors:** Kim Wong, Latasha Ludwig, Oscar Krijgsman, David J. Adams, Geoffrey A. Wood, Louise van der Weyden

**Affiliations:** 1 Wellcome Sanger Institute, Wellcome Genome Campus, Hinxton, Cambridge CB10 1SA, UK; 2 Department of Pathobiology, University of Guelph, 50 Stone Road E., Guelph, ON N1G 2W1, Canada; 3 Netherlands Cancer Institute, Plesmanlaan 121, 1066 CX Amsterdam, The Netherlands

**Keywords:** Dog, Cat, Visceral, Skin, Hemangiosarcoma, Comparative genomics

## Abstract

Angiosarcoma (AS) is a highly aggressive tumor of blood and lymphatic vessels in humans that shares many similarities with spontaneously occurring hemangiosarcoma (HSA) in dogs and cats. To investigate the genetic suitability of HSA as a model for AS, we sequenced ∼1000 cancer genes in 41 cases of HSA and matched germline tissue: 15 canine visceral HSAs, 13 canine skin HSAs and 13 feline skin HSAs. Analysis of visceral HSAs from dogs presenting with concurrent splenic and cardiac neoplasms showed that the tumors were not independent primaries, consistent with the highly metastatic nature of HSA. Comparison of HSA to AS revealed that several driver genes were recurrently mutated in both species, such as *TP53*, *PIK3CA*, *ATRX*, *GRIN2A* and *LRP1B*. Similar to AS, a UV mutational signature was found in a subset of canine cutaneous HSAs and both species show differing mutational profiles between tissue sites. Our characterization of canine and feline HSA demonstrates many important parallels to AS and provides hope that future studies on these cancers will benefit of all three species.

## Abstract (precis)

**Summary:** Analysis of canine and feline hemangiosarcoma reveals many genetic similarities to human angiosarcoma, including recurrently mutated driver genes, differing mutational profiles between tissues and UV mutational signature, in some cases.

## INTRODUCTION

Angiosarcoma (AS) is a rare tumor that accounts for 1-2% of soft-tissue sarcomas in humans, which in themselves comprise <1% of adult malignancies (Lahat et al., 2010). AS arises from the endothelial cells lining the walls of blood or lymphatic vessels and, as such, may occur at any site in the body, including the skin, breast, visceral organs or deep soft tissues. However, the most common presentations are in the skin (typically on the scalp, face and neck) (Lydiatt et al., 1994) or in the breast (as secondary AS, following radiotherapy for breast cancer) (Arora et al., 2014). Known risk factors for AS include radiotherapy, UV light exposure, chemical exposure (polyvinyl chloride, arsenic and thorium dioxide), chronic lymphedema and various familial syndromes (Young et al., 2010). The multifocal and aggressive nature of this tumor results in many patients showing advanced disease at presentation and having a high risk of local recurrence and metastasis, thus resulting in a poor prognosis (30-40% disease-specific survival) (Mark et al., 1996; Fayette et al., 2007; Buehler et al., 2014). The rarity of AS, together with the fact that this tumor type encompasses a heterogeneous group of sarcomas with specific behaviors depending on the primary site (Fayette et al., 2007), has made identification of the genetics underlying the pathogenesis of AS somewhat challenging. The driver genes of AS are beginning to emerge from whole-genome, whole-exome and targeted sequencing studies (Behjati et al., 2014; Murali et al., 2015; Zehir et al., 2017; Painter et al., 2020), yet this is still a disease with significant unmet clinical needs, as prospective studies addressing the various treatment options are limited and the development of new therapies is hampered by a lack of good preclinical models.

Although genetically engineered mouse models have proven useful in understanding the biology and signaling pathways of AS in humans (Dill et al., 2012; Chadwick et al., 2018; Salter et al., 2019), spontaneous hemangiosarcoma (HSA) in companion (pet) animals may represent more relevant clinical models. Dogs, in particular, are relevant models, as they spontaneously develop these tumors, have a larger body size, heterogeneous population, breed predispositions, comorbidities (such as obesity, asthma, hypertension) and shared environmental exposures with humans (LeBlanc and Mazcko, 2020). Given that pet cats share many of these features, they may also represent relevant models. Similar to AS, HSA is an aggressive tumor arising from endothelial cells and, particularly visceral disease, is associated with a high risk of recurrence and/or poor prognosis due to advanced stage of the disease at presentation (because of its multifocal nature and presence of metastases). In addition, AS and HSA exhibit equivalent histopathological features and cell of origin (Fosmire et al., 2004; Kim et al., 2015). HSA represents up to 7% of all canine malignant tumors (Vail and MacEwen, 2000), with visceral organs, particularly the spleen, right atrium/auricle of the heart, and liver, as the most common primary sites. The development of visceral HSA has shown a breed predisposition, with Golden retrievers, boxers and German shepherds being at increased risk (Prymak et al., 1988; Spangler and Culbertson, 1992; Tamburini et al., 2009). Breed disposition suggests that heritable traits contribute to this disease; however, it cannot be excluded that the relatively high frequency of HSA in dogs, compared to humans, might also be due to specific features of the canine microvasculature, and/or differences in hemostasis and inflammation (reviewed in Kim et al., 2015). Metastatic disease of visceral HSA is commonly present early in tumor development, particularly in the lungs, liver, mesentery and omentum. It is not uncommon for dogs to present with concurrent visceral HSA tumors of the spleen and heart (Waters et al., 1988; Boston et al., 2011; Yamamoto et al., 2013), and it is not known whether these represent independent primary tumors or metastatic disease. Dogs can also develop cutaneous HSA, particularly in non-pigmented or light-haired skin (often on the ventral abdomen), with UV radiation proposed to play a causative role (Hargis et al., 1992). Visceral HSA is associated with a significantly worse prognosis than cutaneous HSA (Schultheiss, 2004), owing to local infiltration, rupture of the primary tumor and/or metastases (Brown et al., 1985). In cats, HSA is overall less common, with an incidence of 0.5-2% of all tumors (Smith, 2003), and more commonly found in the skin than the visceral organs, with the latter being relatively rare (Johannes et al., 2007). In both dogs and cats, HSAs involving the subcutis (cutaneous extending into subcutis or subcutaneous form) are more biologically aggressive than the cutaneous form, and more likely to locally recur (Johannes et al., 2007), whereas cats that had aggressive surgical excision of their cutaneous tumors showed good long-term prognosis (McAbee et al., 2005).

Whole-exome sequencing of canine HSAs has previously been performed in two studies, including splenic tumors from a variety of breeds (*n*=20) (Wang et al., 2017) and visceral tumors (spleen, heart and liver) from Golden retrievers (*n*=47) (Megquier et al., 2019). A more recent study performed amplicon-based targeted resequencing of splenic tumors (*n*=50) using a 30-gene ‘HSA panel’ (Wang et al., 2020). However, there were differences in the candidate driver genes identified in each of these studies, suggesting that HSA is a heterogeneous tumor type, which is in agreement with studies of AS (Boichard et al., 2020). Furthermore, there have been no detailed genetic analyses of canine skin HSA [only studies looking at mutations in *PDGFRA*/*B* (Abou Asa et al., 2015) and *c-KIT* (Chen et al., 2016), specifically], and no genetic analyses of feline skin HSA. Thus, we asked whether deep sequencing of a targeted gene panel would provide novel insights into the genetics of canine and feline HSA, and whether they are relevant models of AS in humans. This has the potential to open up avenues for the application of human therapies to these animals, as a number of drugs utilized in veterinary medicine were originally developed for human use (reviewed in Paoloni and Khanna, 2008). Likewise, cancer clinical trials in companion animals have potential applications to human patients, especially when the cancer type is more common in animals. This line of thinking is concordant with the ‘One Medicine’ concept that promotes the view that human and veterinary medicine share many commonalities and both can contribute to the advancement of the other (Schwabe, 1964). This has evolved into the ‘One Health Initiative’ that promotes interdisciplinary collaboration in aspects of health for humans, animals and the environment (https://onehealthinitiative.com/). To be a relevant model of AS in clinical trials, alterations in cancer-associated driver genes are of most interest. Thus, we performed targeted sequencing of ∼1000 cancer-associated genes in paired splenic and cardiac HSA tumors from the same dogs, as well as cutaneous HSA tumors from both dogs and cats.

## RESULTS

### Sequencing of canine and feline HSA

We performed targeted panel sequencing of cancer-associated genes in tumor-germline (matched normal tissue) pairs of 15 primary canine visceral HSA cases, 13 primary canine cutaneous HSA cases and 13 primary feline cutaneous HSA cases. Fourteen of the canine visceral cases were from dogs with concurrent cardiac and splenic HSA lesions (where both lesions were sequenced) and one was a cardiac lesion only. The canine and feline cutaneous cases ranged from dermal to subcutaneous regions of the skin, from different body sites (including the ventral abdomen, limbs and back). A summary of all cases and their signalment data is provided in Table S1. The tissue was sampled by taking cores from formalin-fixed paraffin-embedded (FFPE) blocks. All cores were taken in duplicate, and the DNA was extracted from each core using one of two different methods (detailed in the Materials and Methods) to provide replicates to aid in variant calling. The methods showed a high degree of concordance in variant calling (Fig. S1). Using a list of 1039 human cancer-associated genes in the OncoKB database (https://www.oncokb.org/cancerGenes), we identified the canine and feline orthologs of these genes, where possible, and were able to include a total of 962 and 986 genes for the canine and feline bait libraries, respectively (Table S2). To explore the cancer gene mutational landscape of these tumors, we generated profiles of somatic point mutations, multi-nucleotide variants, indels and somatic copy number alterations (SCNAs) for these genes. Complete lists of the somatic mutations identified in each sample are provided in Table S3.

### Concurrent canine splenic and cardiac HSA lesions are not independent tumors

**Fig. 1.**
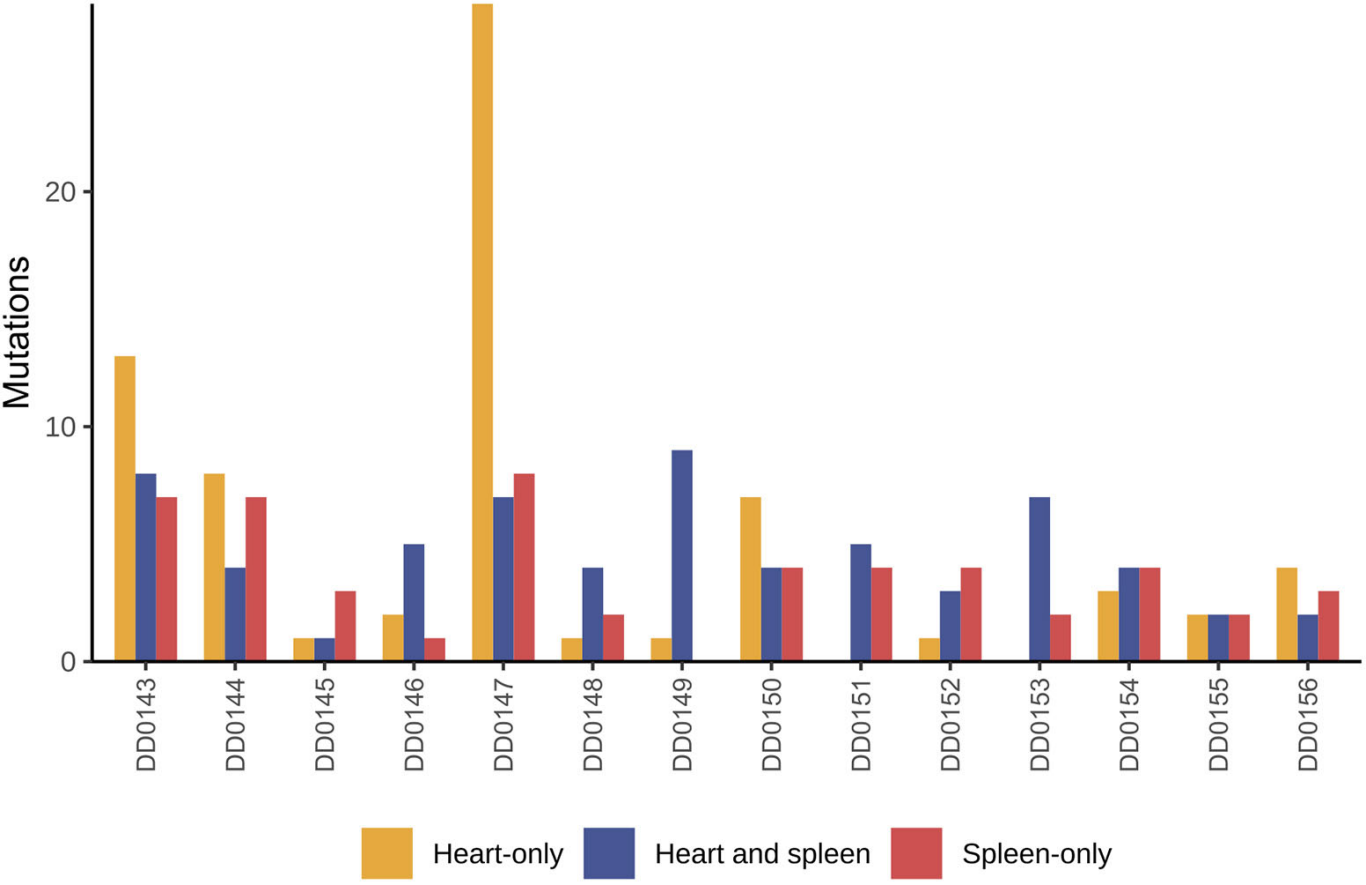
**Shared and unique somatic mutations in canine cardiac and splenic hemangiosarcoma (HSA) from the same patient.** Shown here are the number of unique and shared mutations [defined as mutations in the same genomic position with the same nucleotide change(s)] in tumors from dogs that presented with concurrent cardiac and splenic HSA.

Post-mortem studies on dogs with clinical signs of cardiac (right atrium) HSA have commonly reported the presence of concurrent splenic HSA [17/51 (33%) cases (Yamamoto et al., 2013) and 9/31 (29%) cases (Boston et al., 2011)]. Conversely, studies on dogs with clinical signs of splenic HSA have reported the presence of concurrent cardiac HSA [6/25 cases (24%) (Waters et al., 1988) and 2/23 (8.7%) cases (Boston et al., 2011)]. However, it is not known whether these represent independent/synchronous primary tumors or metastatic disease. To answer this question, we obtained tumor samples from 14 dogs with concurrent splenic and cardiac HSA lesions. The paired samples from each dog had shared mutations (defined as mutations in the same genomic position with the same nucleotide change; Fig. 1), and almost all of these mutations were not seen in tumors from other dogs. For example, although almost all tumors from different dogs had different *TP53* mutations, concurrent cardiac and splenic tumors from each dog shared the same *TP53* mutation (Fig. S2); four of the concurrent HSAs had two identical *TP53* mutations. It is highly unlikely that this pattern of *TP53* mutations occurred by chance in independent tumors, in agreement with the overall overlap of mutations shown in Fig. 1. Thus, we can conclude that the concurrent lesions do not represent two independent tumors, and it is more likely that one is a metastasis of the other.

### Cancer gene mutation landscape of canine visceral HSA tumors

**Fig. 2.**
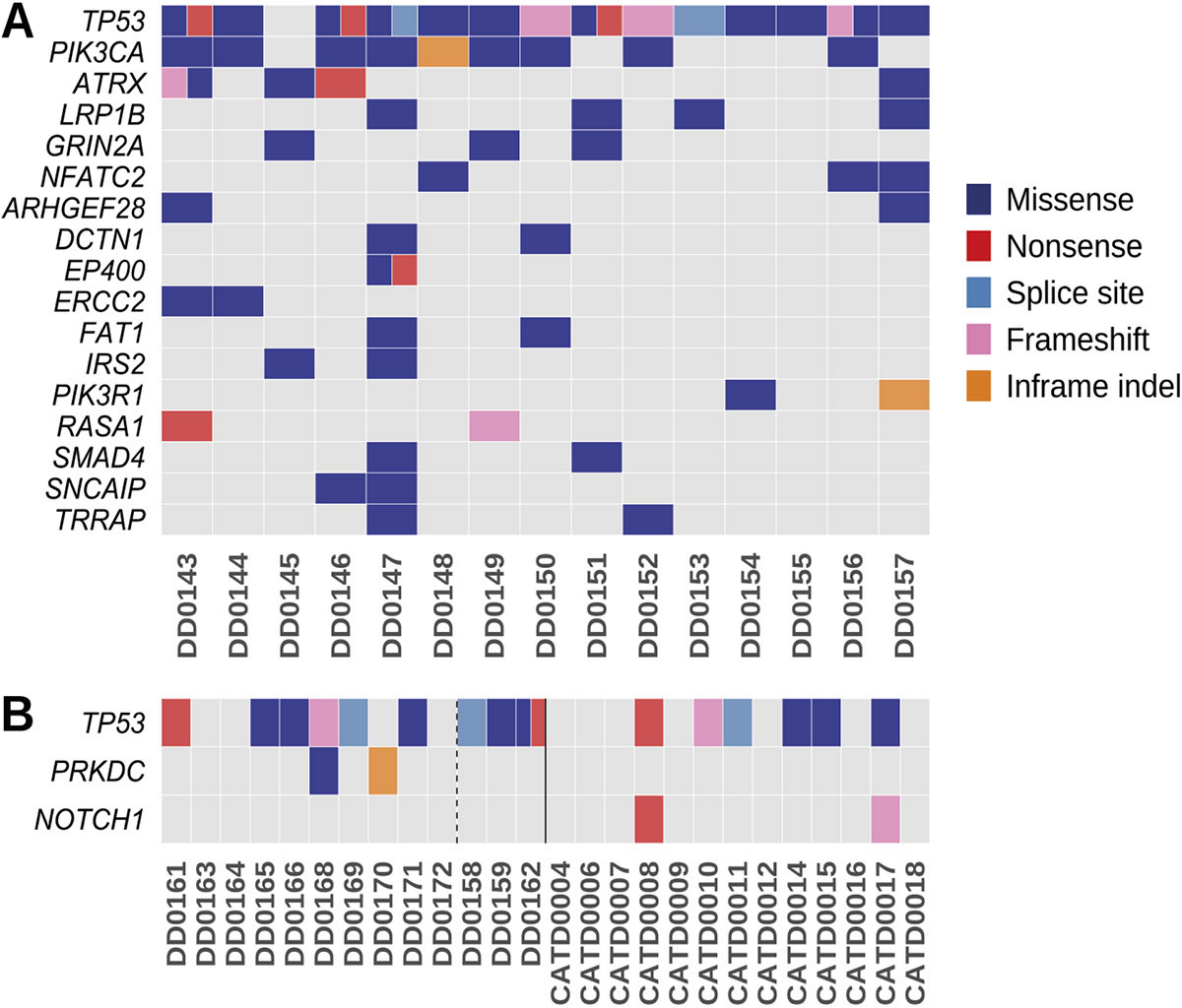
**Cancer gene landscape of canine visceral, canine cutaneous and feline cutaneous HSA tumors.** (A,B) Mutations in canine visceral HSA (A) and canine and feline cutaneous HSA (B). Canine samples are left of the solid line; feline, right. Canine cutaneous HSA samples with a UV mutational signature are right of the dashed line. Mutations in visceral HSA tumors were present in either the heart sample, spleen sample or both. Shown are genes mutated in two or more samples, excluding those with a UV mutational signature. A full summary of the cases analyzed in this study is provided in Table S1.

For clarity, and as we have shown that the cardiac and splenic tumors are not independent tumors, they will be discussed together and referred to as visceral HSA. Our analysis of canine visceral HSAs found that the tumor suppressor *TP53* was the most frequently mutated gene (14/15 cases, 93%; Fig. 2A); all but one of the mutations occurred in the DNA-binding domain, which is the most frequently mutated domain in *TP53* in human cancers (Laptenko and Prives, 2006). Interestingly, six cases had multiple mutations in the gene (Fig. S2). The oncogene *PIK3CA* was the second most frequently mutated gene in canine visceral HSA (9/15 cases, 60%; Fig. 2A), with the majority of mutations (6/9) identified as H1047R in the kinase domain (Table S3A and Fig. S2). The PIK3CA protein is highly conserved between human and dog (99.8% identity), including amino acid H1047. PIK3CA H1047R is a hotspot missense mutation in many human cancers that increases the catalytic activity of PI3K (Samuels et al., 2004). Interestingly, one canine HSA (DD0144) with a *PIK3CA* mutation also carried a mutation in another class I PI3K gene, *PIK3C2G*, and two cases (DD0154, DD0157) without mutations in *PIK3CA* had mutations in its regulatory subunit, *PIK3R1* (Table S3A). The next most frequently mutated genes in the canine visceral HSAs were the tumor suppressor genes *ATRX* (with DD0143 carrying one independent and two linked mutations in the gene) and *LRP1B* (4/15 cases, 26%), followed by *GRIN2A* and *NFATC2* (3/15 cases, 20%; Fig. 2A). Finally, there were 11 genes mutated in 2/15 (13%) of cases, specifically, *ARHGEF28*, *DCTN1*, *EP400*, *ERCC2*, *FAT1*, *IRS2*, *PIK3R1*, *RASA1*, *SMAD4*, *SNCAIP* and *TRRAP* (Fig. 2A).

### Cancer gene mutation landscape of canine cutaneous HSA tumors

Similar to visceral HSAs, the most frequently mutated gene in the canine skin HSA cohort was *TP53* (9/13 cases, 69%; Fig. 2B). However, in contrast to visceral HSAs, the only other recurrently mutated gene, when excluding samples with a UV signature, was the protein kinase *PRKDC* (2/13 cases, 15%). Interestingly, in three of the canine skin HSA samples (DD0158, DD0159, DD0162), we identified an elevated mutation rate and the COSMIC mutational signatures SBS7a and SBS7b (Fig. S3), which have been found predominantly in skin cancers and have a proposed etiology of UV light exposure (Alexandrov et al., 2020). These tumors were located on the hindlimb and ventral abdomen of these dogs, which are sun-exposed sites (if the dog ‘sun bathes’ belly up), thus suggesting a potential role for UV light exposure in the development of some cutaneous canine HSAs. This is consistent with that reported for face and scalp AS in humans (Painter et al., 2020; Boichard et al., 2020). As a result of exposure to UV light, these three canine cases have a significantly increased number of somatic mutations relative to the other cases, which confounds driver gene identification, as the majority of the recurrent mutations are likely to be passenger mutations. However, if these tumors are taken into account, then other recurrently mutated genes in the skin HSAs include *RELN* and the tumor suppressor gene, *PTPRD* (4/13 cases, 30%; 3/4 cases had a UV mutational signature; Table S3A). Other recurrently mutated genes included *PIK3CA*, *LRPB1*, *PLCG2*, *KMT2D* and *ERBB4* (3/13 cases, 23%; 2/3 cases had a UV mutational signature; Table S3A). Finally, *SMARCA1* and the tumor suppressor gene *PTEN* were also recurrently mutated (2/13 cases, 15%; 1/2 cases had a UV mutational signature; Table S3A).

### Cancer gene mutation landscape of feline cutaneous HSA tumors

Similar to canine skin HSA tumors, the most recurrently mutated gene in the feline skin HSA cohort was *TP53* (6/13 cases, 46%; Fig. 2B; Table S3B). However, the only other recurrently mutated gene was the tumor suppressor gene *NOTCH1* (2/13 cases, 15%), with tumors harboring nonsense and frameshift mutations. *NOTCH1* was not mutated in any canine skin HSA cases, although one canine visceral HSA case harbored a frameshift mutation in *NOTCH1*. There were additional similarities to canine skin HSA, as *RELN*, *PIK3CA* and *KLHL6* were mutated in both species (in one case each, if excluding the canine cases with a UV mutational signature; Table S3).

### Analysis of SCNAs

**Fig. 3.**
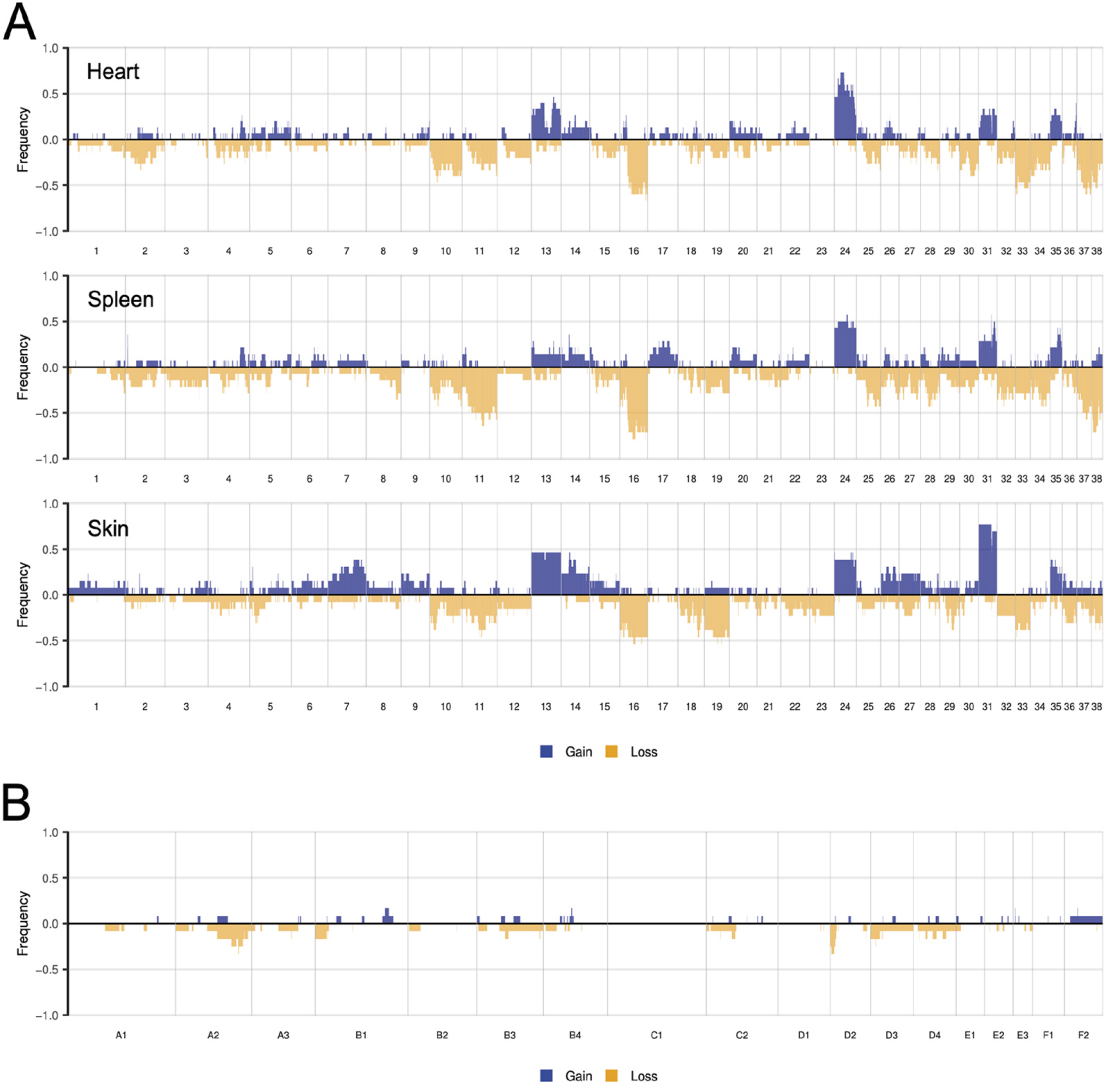
**Somatic copy number alterations (SCNAs) of canine visceral, canine cutaneous and feline cutaneous HSA tumors.** (A,B) Penetrance plots showing the frequency of SCNAs in canine cardiac (top; *n*=15), splenic (middle; *n*=14) and skin HSAs (bottom; *n*=13) (A), and feline skin HSAs (*n*=13) (B).

We next analyzed the copy number profiles of canine visceral, canine cutaneous and feline cutaneous HSA. The frequencies of SCNAs in each cohort are shown in Fig. 3, and representative copy number profiles of individual samples are shown in Fig. S4. Canine visceral HSAs had substantial chromosomal gains and losses, the most frequent of which were gains along chromosomes 13, 14, 24, 31 and 35, and losses along chromosomes 10, 11, 16, 33, 37 and 38 (Fig. 3A). This pattern is similar to that identified in primary canine visceral HSA in a study using oligonucleotide array comparative genomic hybridization (CGH) (Thomas et al., 2014), in which copy number gain of chromosomes 13, 24 and 31 and loss of chromosome 16 were most prevalent. We also noted differences in the penetrance of recurrent copy number gains and losses, which may be attributed to the differences in the sensitivities of the technologies used and/or differences in SCNA penetrance amongst breeds (Thomas et al., 2014). The cardiac and splenic tumors had very similar copy number profiles, lending additional support to our assertion that concurrent cardiac and splenic tumors are not independent tumors. Although genome-wide copy number profiling has previously focused on visceral HSA, we were able to compare visceral and cutaneous HSA and observe differences in the penetrance of copy number gains of chromosome 13, 14, 24 and 31 (Fig. 3). However, as the visceral and cutaneous HSA samples were obtained from a variety of breeds, we are unable to attribute these differences to tissue site or breed differences. It would be of interest, in future studies, to use larger cohorts of cutaneous HSA to study these differences. The feline skin HSAs had relatively few SCNAs compared to the canine skin HSAs, and the most penetrant SCNAs were smaller regions of chromosomes A2 and D2 (Fig. 3B).

### Analysis of germline sequence data for pathogenic mutations

In addition to our analysis of somatic mutations, we looked for putative pathogenic germline variants in the canine and feline orthologs of established human AS susceptibility genes (Cao et al., 2019), specifically, *ERCC2*, *IDH1*, *IDH2*, *PIK3CA*, *POT1*, *PTEN*, *RB1*, *TP53* and *XPC.* The AS susceptibility genes *POLH* and *PTHR1* were not included in OncoKB and thus were not part of our targeted gene panel (see Materials and Methods). We identified missense variants in *ERCC2* (six dogs), *IDH1* (three dogs) and *IDH2* (two dogs; Table S4A). We also identified a nonsense mutation in *TP53* in two dogs. However, the location of this variant (C>T, chromosome 5 at position 32,565,554) falls within only one of the three canine *TP53* transcripts annotated in Ensembl (v98, TP53-202, exon 1), and it is only represented at very low levels in RNA-sequencing (RNA-seq) data from canine visceral HSA samples (Megquier et al., 2019). In addition, the gene model may be erroneous, as there is evidence of at least one additional intron/exon upstream of exon 1 in TP53-202 (Fig. S5). Finally, we identified missense variants (at five different locations) in *XPC* in 15 dogs; however, as we found that a number of other sites in *XPC* were not true variants but instead reference genome CanFam3.1 errors that have been corrected in CanFam4, it is likely that the majority of these are also due to reference genome errors. In the cat germlines, we identified missense mutations in *ERCC2*, *RB1*, *IDH1*, *IDH2*, *POT1*, *TP53* and *XPC* (Table S4B). In three cases, *RB1* had an additional in-frame deletion. It would be of interest to further investigate the variants in these orthologs of established human AS predisposition genes to determine whether they play a role in germline predisposition to HSA in dogs and cats.

### Comparative analysis with AS in humans

**Fig. 4.**
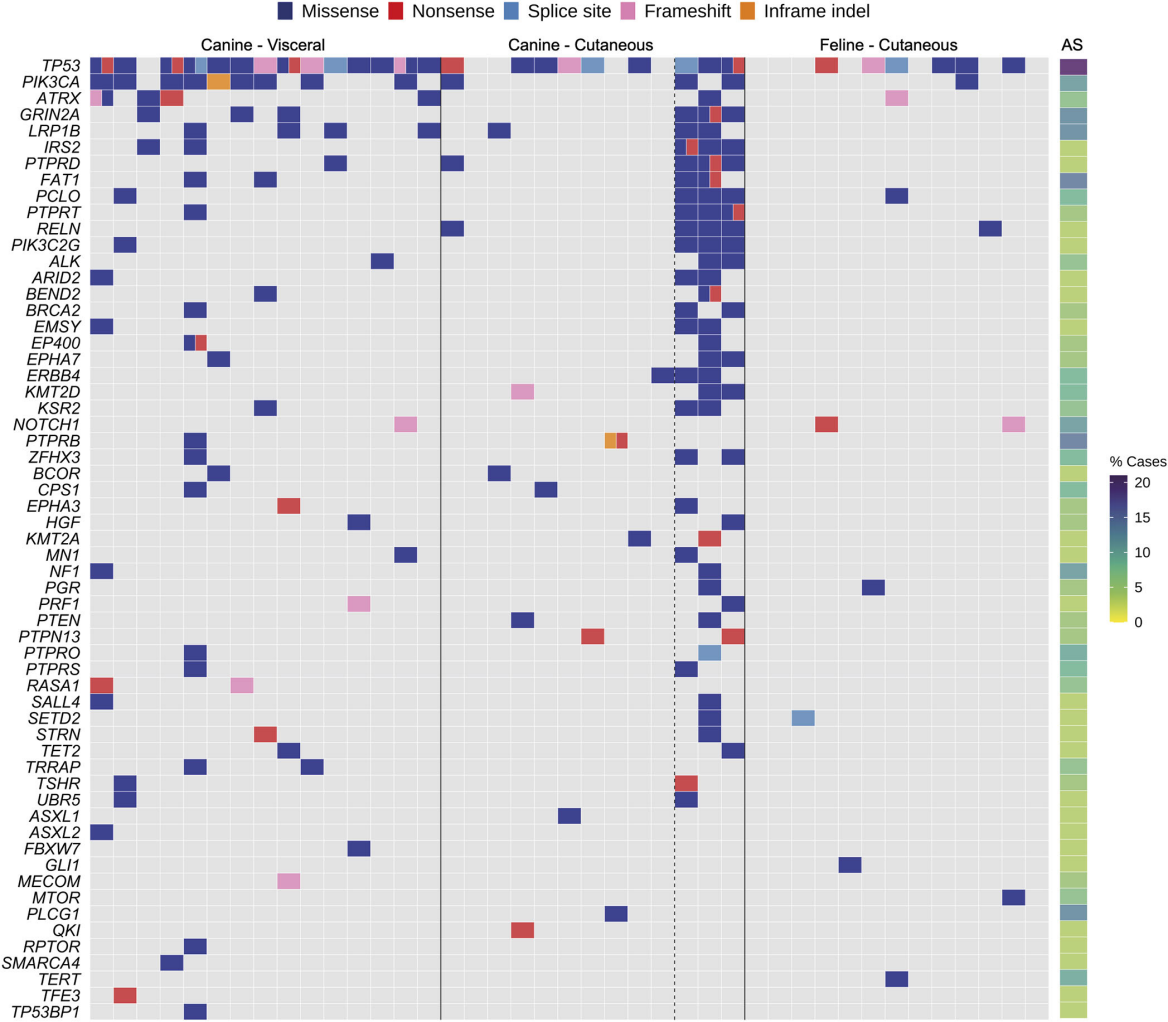
**Comparative mutational landscape of human angiosarcoma (AS) and canine and feline HSA tumors.** Shown are genes mutated in at least two of the three species. The human AS data from 62 samples were obtained from the Angiosarcoma Project (Painter et al., 2020). The genes mutated in human AS are represented as a percentage of total cases.

AS is a heterogeneous tumor type, with differences in the relative frequencies of the mutated genes reported between studies (Behjati et al., 2014; Murali et al., 2015; Zehir et al., 2017; Painter et al., 2020). For the comparison of canine and feline HSA to human AS, we used the data from the Angiosarcoma Project (July 2020; 62 patients) (Painter et al., 2020). As some patients had samples from multiple time points, we selected one sample per patient for the comparison (Materials and Methods; Table S5). In all three species, the tumor suppressor gene *TP53* is the most recurrently mutated gene (Fig. 4), although the frequency is higher in dogs (69-93%) and cats (46%) than in humans (21%). Interestingly, 6/28 (21%) canine HSAs had two *TP53* mutations compared to 3/62 (4.8%) human HSAs. The oncogene *PIK3CA* is recurrently mutated in canine HSA [both in our study and others (Megquier et al., 2019)] and in AS patients (9/62 patients, 10.8%; Fig. 4), although it is exclusively mutated in AS from the breast (9/9 patients) (Painter et al., 2020). In our cohort, we found that more visceral HSA cases had *PIK3CA* mutations (9/15, 60%) than skin HSA samples (3/13, 23%); Fig. S2), which may also explain why *PIK3CA* was only mutated in a single feline skin sample (1/13, 7.6%). The putative tumor suppressor gene, *LRP1B* (Fig. 4), is also mutated in both human AS and canine HSA.

The transcriptional regulator *ATRX* is recurrently mutated in human AS (5/62 cases, 6%; Fig. 4) (Painter et al., 2020) and immunohistochemical staining revealed that a subset of patient samples have loss of ATRX expression [7/118 cases, 6% (Panse et al., 2018)]. Loss of ATRX expression was more frequently observed in tumors located in deep soft tissues than in other body sites, and associated with a significantly worse event-free survival (Panse et al., 2018). We report, for the first time, that *ATRX* is also recurrently mutated in canine HSA (4/15 visceral samples and 1/13 skin samples) (Fig. 4). Previously, mutation of *ATRX* had only been reported in 1/47 canine visceral HSA samples (Megquier et al., 2019). Interestingly, loss of *atrx* was found to cooperate with p53 deficiency in promoting the development of sarcomas in zebrafish (Oppel et al., 2019), and in our cohort, 3/4 (75%) canine HSAs with *ATRX* mutations also had *TP53* mutations (Fig. 4; Fig. S2). We also provide the first reports of recurrent mutation of *FAT1*, *GRIN2A*, *PLCO* and *RELN* in canine HSA (with the latter two genes also mutated in feline HSA), which are also recurrently mutated in AS (Fig. 4). Several receptor-type protein tyrosine phosphatases (PTPRs) are recurrently mutated in AS; we identified mutations in *PTPRB*, *PTPRD*, *PTPRO*, *PTPRS* and *PTPRT* (Fig. 4) in canine HSA, while previously, only mutations in *PTPRD* have been reported (Megquier et al., 2019).

The genetic landscape of feline HSA has not been reported previously. Of the genes in our feline gene panel, we found protein-altering mutations in 21 genes (Table S3B). Both *TP53* and *NOTCH1* were recurrently mutated in feline HSA and in human AS (Fig. 4). In addition, we found mutations in other genes that were mutated in feline HSA and recurrently mutated in AS (*ATRX*, *GLI1*, *MTOR*, *PCLO*, *PGR*, *PIK3CA*, *RELN*, *SETD2* and *TERT*; Fig. 4) or mutated in one AS sample (*BIRC3*, *CALR*, *LATS2*, *PAX3* and *TEK*) (Painter et al., 2020). Thus, 16/21 (76%) of the genes with protein-altering mutations in feline skin HSA are also mutated in AS.

When considering only AS of the head/face/neck/scalp (HFNS) in the Painter et al. (2020) cohort, the most frequently mutated gene is *TP53* (9/19 patients, 47%), as was the case in the canine and feline skin HSA samples (Fig. 4). Importantly, *NOTCH1* is frequently mutated in AS of the HFNS (5/19 samples, 26%) and was also recurrently mutated in feline skin HSA (one frameshift mutation and one nonsense mutation) (Fig. 4). Critically, decreased NOTCH1 expression by immunohistochemistry has been reported in AS (29/123 cases, 24%) and is statistically associated with a cutaneous site of origin (Panse et al., 2018). Additionally, inhibition of NOTCH signaling induces the development of malignant vascular tumors in mice (Liu et al., 2011; Dill et al., 2012). Interestingly, however, *NOTCH1* mutations were not seen in any of the canine skin HSA samples. Yet, it is worth mentioning that decreased NOTCH2 expression has been reported in AS (16/103 cases, 16%) (Panse et al., 2018), *NOTCH2* mutations have been reported in skin and HFNS AS (Painter et al., 2020), and *NOTCH2* mutations were seen in 2/13 of the canine skin HSA samples (albeit that both cases had a UV mutational signature and elevated mutation rate). Taken together, this would suggest a tumor-suppressive role for the NOTCH signaling pathway in the pathogenesis of AS. There are other parallels between canine skin HSA and human skin AS. For example, a UV mutational signature was found in 3/13 canine skin samples (Fig. 1; Fig. S3), and AS of the HNFS is also associated with a strong UV mutational signature (Painter et al., 2020), suggesting that, for AS of HNFS and canine cutaneous HSA, UV-light-induced DNA damage may be a causative factor.

### Identification of actionable mutations

By specifically sequencing canine and feline orthologs of human cancer genes and comparing altered genes with AS, we were able to identify several potential actionable mutations. In all three species, we observed mutations within the PI3K/AKT/mTOR pathway, including *PIK3CA*, *PIK3C2B*, *PIK3C2G*, *PIK3R1*, *MTOR* and *PTEN*. The PI3K/AKT/mTOR pathway is an important intracellular signaling pathway responsible for regulation of the cell cycle, and there are a range of PIK3 inhibitors in clinical trials, either dual PI3K/mTOR inhibitors, pan-PI3K inhibitors or isoform-specific inhibitors (reviewed in Yang et al., 2019). For example, clinical trials of the PI3K inhibitor, ZSTK474, showed prolonged stable disease for sarcoma patients (Lockhart et al., 2013), and several groups have reported on the potential efficacy of PI3K inhibitors in sarcoma mouse and zebrafish models, such as BKM120 (Buparlisib) in undifferentiated pleomorphic sarcoma (Kim et al., 2012), and BEZ235 in well-differentiated liposarcoma (Gutierrez et al., 2011) and chondrosarcoma (Zhang et al., 2013). Furthermore, a novel dual PI3K/mTOR inhibitor, VDC-597, showed dose-dependent inhibition of both Akt1 and 4eBP1, reduction of proliferation, migration and VEGF production, and promotion of tumor cell apoptosis in three canine HSA cell lines (Pyuen et al., 2018). *NOTCH1* is recurrently mutated in human AS and feline HSA, and mutated in one canine visceral HSA sample, and there are many clinical trials using different types of drugs targeting NOTCH, including receptor/ligand antibodies, gamma secretase inhibitors (GSIs) and NOTCH transcription complex inhibitors, with a Phase III trial of the GSI Nirogacestat currently underway (reviewed in Moore et al., 2020). In addition, as a subset of HNFS AS (Painter et al., 2020) and cutaneous HSA tumors harbor a high mutation rate and a strong UV mutational signature, immune checkpoint inhibition may be beneficial in these patients. Indeed, pilot clinical studies assessing the effectiveness of therapeutic antibodies against canine PD-L1 and PD-1 for advanced spontaneous cancers in dogs have shown some success in canine oral melanoma and undifferentiated sarcoma patients (Maekawa et al., 2017; Igase et al., 2020). Finally, *PIK3CA* has been shown to be associated with sensitivity to ionizing radiation when mutated (Yard et al., 2016); thus, given the frequency of *PIK3CA* mutations in breast AS (Painter et al., 2020) and canine visceral HSA, radiotherapy may also be beneficial in these patients.

## DISCUSSION

In our cohort, recurrently mutated genes found in canine HSA included *TP53*, *PIK3CA* and *LRP1B*, which is consistent with previous reports in canine HSA (Megquier et al., 2019; Wang et al., 2020) and in AS. Critically, despite the small cohort size, we have also identified cancer-associated genes that are recurrently mutated in AS, but not previously reported as such in canine HSA, including *ATRX*, *FAT1*, *GRIN2A* and *RELN*, further strengthening the support for canine HSA as a relevant model for AS in humans. In addition, targeted sequencing of canine cutaneous HSA revealed strong parallels with the genetics of AS of HFNS, including the identification of a UV mutational signature. Sequencing of feline cutaneous HSA for the first time also revealed parallels to both canine cutaneous HSA and human AS. Genetic parallels between canine/feline HSA and AS, in addition to the clinical and histopathological parallels, makes dogs and cats attractive as relevant models for AS in humans.

The tissue site in which the AS arises plays a strong role in the biology of the disease and thus the prognosis of the patient (Fayette et al., 2007), and the same is true for canine and feline HSA. Visceral HSA is associated with a poorer prognosis than cutaneous HSA [with respect to cutaneous HSA lesions that can be completely excised with clear margins, which have a good to fair prognosis (Schultheiss, 2004)]. This may be partly attributed to the relative accessibility of these tissues for diagnosis and treatment (such as skin versus visceral). However, it could also be attributed to the differences in the mutational profile of the tumors at each site. For example, *PIK3CA* and *KDR* mutations are predominantly found in AS tumors of the breast (7/7 and 9/11 samples with mutations in these genes, respectively, were from breast tissue) (Painter et al., 2020). Having sequenced orthologs of human cancer genes in canine HSAs from multiple tissue locations, specifically visceral and skin, for the first time, we are able to see that, similar to AS, there are differences in the mutational landscape in canine HSA from different tissue sites. First, despite similar cohort sizes, the visceral HSA cohort has more genes affected by protein-altering mutations than the cutaneous cohorts; 21 and 27 genes in the feline HSA and canine HSA cohorts, respectively (excluding samples with a UV mutational signature) relative to 113 genes in the canine visceral cohort. Second, some of the canine cutaneous samples had a UV mutational signature, which was not seen in the canine visceral HSA samples. Third, although both the visceral and cutaneous samples have some mutated genes in common, such as *TP53* and *PIK3CA*, there are some tissue-specific differences. For example, *PIK3CA* was mutated in 9/15 visceral HSA samples (with 6/9 at amino acid hotspot position 1047) but only 3/13 skin samples (with 1/3 at position 1047). Finally, there are also differences between SCNAs in different tissues. For example, gains across chromosome 24 are seen in a higher proportion of visceral cases relative to skin cases, yet a higher proportion of gains across chromosomes 13, 14 and 31 are seen in the skin cases relative to the visceral cases. Further studies are required to confirm whether these differences are inherent to the tumor tissue site or due, in part, to differences in breeds.

In addition to tissue-specific differences in the mutational profiles, it is important to note that there are also species-specific differences. For example, the tumor suppressor gene *POT1* is mutated in 11/62 (17.7%) of AS cases (seven from HFNS and four from breast) (Painter et al., 2020); however, we, and others (Megquier et al., 2019), have not observed any somatic mutations of *POT1* in canine HSA. Similarly, the tumor suppressor gene *ROBO1* is mutated in 8/62 AS cases (six from HFNS and two from breast) (Painter et al., 2020), but not in this or previous HSA studies (Megquier et al., 2019). The significance of this is uncertain at this stage, as mutations in these genes may be found in larger cohorts, if they are low penetrance driver genes in HSA. Alternatively, given the tissue-specific preferences of some AS driver genes, sequencing of canine/feline HSAs from other tissue sites may reveal these as driver genes in HSA.

Retrospective post-mortem studies on dogs that presented with clinical signs of cardiac HSA have reported the presence of concurrent splenic HSA and vice versa (Waters et al., 1988; Boston et al., 2011; Yamamoto et al., 2013). However, some dogs that present with multiple splenic HSAs have no involvement of the heart or any other primary site, and some dogs that present with cardiac HSAs have no gross metastases in the spleen or other primary sites (Yamamoto et al., 2013). Thus, there is strong evidence that primary HSA can arise in either the heart or spleen; however, it is unclear whether the common occurrence of concurrent splenic and cardiac HSA represents two independent primary tumors or one metastasis originating from the other. We showed that paired (concurrent) splenic and cardiac HSAs from each dog shared mutations and had similar SCNAs, which indicates they are not independent primaries and thus favors the likelihood that one represents metastasis from the other site. This is consistent with AS, in which a significant number of patients have metastases at the time of presentation, with one study reporting metastases at presentation in 26/81 (32%) AS patients (Buehler et al., 2014). More extensive sequencing across the exome or genome is required to decipher the evolutionary histories of the canine splenic or cardiac HSA and determine which are the metastases. However, these findings have significant clinical implications, as early detection of HSA by screening blood samples (as is in development at the University of Minnesota; ‘Shine On’ Project, https://vetmed.umn.edu/centers-programs/clinical-investigation-center/current-clinical-trials/early-detection-target-hemangiosarcoma-cells-dogs-shine-project), followed by removal of the spleen or right atrium to prevent an aggressive HSA from growing and metastasizing, is much more likely to be successful (curative) if there is not a high risk of dual primaries forming.

A limitation of our study is the small cohort size. It would have been interesting to determine whether some of the genetic alterations we observed are associated with parameters such as treatment response and patient survival; however, because the genetic alterations identified are very heterogeneous, such analysis would require a significantly larger cohort of HSA from each tissue site. In addition, given that we did not find any protein-altering single-nucleotide variants (SNVs) or indels in our gene panel in 3/13 feline cases (CATD0004, CATD0006, CATD0018), or a UV mutational signature in any feline case, it would suggest that future studies may have to use substantially larger cohorts in order to be able to identify rare driver genes and definitively determine whether a UV mutational signature exists in a proportion of feline cutaneous HSA tumors. However, it should be noted that very few synonymous mutations (<3), all with low variant allele frequencies, were detected in these three samples, and thus the possibility remains there was a lack of tumor cells present in the sample biopsy (core) under the surface; the tumor can only be assessed at the surface of the block, and whether the tumor decreases in size or cellularity deeper in the block cannot be predicted. Nevertheless, we have been able to find recurrently mutated genes that are shared between canine/feline HSA and AS that have not previously been reported, thus expanding the list of putative driver genes in HSA, and our analysis of HSA from different tissue sites has demonstrated that tissue-specific preferences in the mutational profile exist in canine HSA as in AS. This further cements canine HSA as a relevant model of AS in humans, and offers feline HSA as an attractive additional candidate model. Thus, dogs and cats with HSA can aid in the identification of both key driver genes at different tissue sites and potential therapies in clinical trials. Indeed, pet dogs with spontaneously developed cancer have assisted the translation of several new therapies for human cancer through the use of clinical trials that can inform preclinical drug development as well as investigate novel therapeutic approaches (as reviewed in Paoloni and Khanna, 2008; LeBlanc and Mazcko, 2020). Importantly, the dogs and cats with HSA may also potentially benefit from any therapies that show results in AS patients, in keeping with the One Health Initiative.

## MATERIALS AND METHODS

### Canine and feline sample collection and DNA extraction

The samples consisted of FFPE canine and feline tissues submitted to the Ontario Veterinary Clinic in the Department of Pathobiology at the University of Guelph (Canada) as part of routine diagnostic procedures with the owner's consent. The use of the samples adhered to Nagoya Protocol guidelines. The cases were from 1998 to 2019 and were selected based on the availability of matched FFPE normal (healthy) tissue from the same animal (which, in some cases, was adjacent to the tumor) and from a range of breeds. Additionally, the canine visceral HSA samples were selected for cases in which the animal had concurrent splenic and cardiac HSA lesions available for sampling (*n*=15), and the canine skin (*n*=15) and feline skin (*n*=15) samples were selected to cover different tumor locations within the skin (dermis and subcutaneous). A summary of the cases is provided in Table S1, including signalment details such as the breed, sex and age at diagnosis. All tumor and normal tissue samples were obtained as 0.6 mm or 1 mm diameter FFPE cores taken in duplicate (both cores were adjacent in 26/29 canine visceral, 10/13 canine skin and 11/13 feline skin HSAs), with different DNA extraction protocols to theoretically reduce our false-negative rate during variant calling. DNA was extracted from one set of duplicate cores using a QIAamp DNA FFPE Tissue Kit (Qiagen) and a GeneRead DNA FFPE Kit (Qiagen) for the other set, according to the manufacturer's instructions. The latter kit includes an enzymatic removal of cytosine deamination artefacts. The performance of these kits has previously been compared (Bonnet et al., 2018). In addition, six DNA samples that were extracted by the QIAamp DNA FFPE Tissue Kit were divided in half, and one half subsequently processed with the NEBNext FFPE DNA Repair v2 reagents (supplied by New England Biolabs), according to the manufacturer's instructions. The mix contains enzymes formulated to repair DNA damage such as deamination of cytosine and base oxidation.

### Bait design

For gene panel design, we started with the 1039 human cancer genes in the OncoKB database (7 May 2019 update) and the *TERT* promoter region. A list of canine and feline orthologs of these genes was obtained from Ensembl (v98). Agilent SureSelect bait libraries for exon target capture of these genes (and up to 125 bp flanking sequence) and 500 bp upstream of the *TERT* transcription start codon were designed against the canine reference genome CanFam3.1 (Hoeppner et al., 2014) (ELID: S3250944) and the feline reference genome FelCat9.0 (Buckley et al., 2020) (ELID: S3250994). For genes with a ‘one-to-many’ orthologous relationship, all orthologs were included in the bait design. For human cancer genes without a canine or feline ortholog annotated in Ensembl, the canine or feline gene with the same gene symbol was included for bait design. We were able to design baits to include a total of 962 and 986 genes for the canine and feline bait libraries, respectively (Table S2).

### Library preparation and sequencing

Sequencing libraries were prepared from the FFPE-extracted DNA using a NEBNext Ultra II DNA Library Prep Kit (New England Biolabs), according to the manufacturer's instructions. Unique dual index tags were applied, and the samples were amplified by PCR using the KAPA HiFi Kit (KAPA Biosystems) for a minimum of eight cycles. The libraries were quantified using Accuclear dsDNA Quantitation Solution (Biotium), pooled (12-plex) in an equimolar fashion and hybridized with the baits overnight. The multiplexed samples were paired-end sequenced using the NovaSeq platform (Illumina) to generate 101 bp reads.

### Sequence data processing and quality control

Sequencing reads from canine and feline samples were aligned to the CanFam3.1 and Felis_Catus_9.0 reference genome, respectively, using BWA-MEM (Li, 2013), and PCR duplicates were marked using Biobambam2 bammarkduplicates2 (v2.0.146) (Tischler and Leonard, 2014). After excluding samples with quality issues such as low coverage of target regions, excessively small library insert size or contamination, there were 14 canine cases with concurrent splenic and cardiac HSA (and one case with a cardiac HSA only), and 13 canine and 13 feline skin HSAs (Table S1). The median sequence coverage of targeted regions was 318- and 291-fold for these canine and feline samples, respectively, when PCR duplicates were excluded.

### Identification of somatic and germline variants

MuTect (v1.7) (Cibulskis et al., 2013) was used to identify somatic SNVs. Default parameters were used, with the exception of a minimum base quality score requirement of 30 and a maximum of four alternative alleles allowed in a matched normal sample. MAC (v1.2) (Wei et al., 2015) was used to identify multi-nucleotide variants from MuTect output. Small indels were identified using Strelka2 (v2.9.10) (Kim et al., 2018) using default parameters. The Pisces variant caller (v5.2.10.49) (Dunn et al., 2019) was also used for variant calling, in somatic mode for tumor samples and germline mode for normal samples, requiring a minimum base quality of 30, minimum mapping quality of 10 and PCR duplicates filtered. The raw somatic calls from Pisces were used for variant-filtering purposes only, as described below. The germline variants were filtered using the default Pisces filters.

The Variant Effect Predictor (McLaren et al., 2016) and Ensembl gene models (v98) were used to predict the consequences of base changes and indels on proteins. For genes with multiple transcripts, the canonical transcript, as defined by Ensembl, was used to determine the variant consequence, except in the case of canine *TP53*, for which TP53-203 was used rather than the canonical transcript TP53-202. Variants called in canine samples were compared to variants in dbSNP (v151), the Dog Genome SNP Database (DoGSD, v2) (Bai et al., 2015) and 722 canine whole-genome sequences (Plassais et al., 2019). To remove false somatic variant calls, those found in ≥1% of the 722 canine genomes were removed. Feline variant calls were compared to variants in dbSNP (v140) and 54 feline genomes (Buckley et al., 2020).

We identified C>A oxidation artefacts by comparing the mutation spectra from our samples to COSMIC mutational signatures (v3, SBS45 and SBS52) (Alexandrov et al., 2020) and applied a variant allele frequency (VAF) and sequence-context-specific filter. A C>A variant was classified as a false positive if it had a VAF≤0.1 and occurred in the sequence context CCN or TCN (or sequence context NGG or NGA for G>T variants). As DNA sequence data from at least two cores were available for most samples, we were able to leverage this when filtering the somatic variant calls further. Knowing that DNA from FFPE tissue contains artefactual C>T variants from cytosine deamination, we defined a high-confidence C>T variant as one with 10 or more supporting reads and a VAF>0.05. Other SNV types were classified as high-confidence calls if there were eight or more supporting reads and a VAF≥0.04. Any variant that did not meet one of these criteria was discarded, unless the same variant was called in a replicate sample with high-confidence. Indels with a VAF<0.1 were discarded. Finally, a somatic SNV or indel called by MuTect was determined to be a false positive if the variant was found in the raw Pisces calls in at least 10% of samples (with all VAFs<0.05 or all VAFs≥0.05) in which MuTect did not make a call. Finally, if MuTect made a high-confidence variant call in one replicate and not the other, the false negative was rescued if it was found by the Pisces variant caller.

Replicate samples (described above) were used to compare the level of artefacts present in DNA isolated using the QIAamp DNA FFPE Tissue Kit and the GeneRead DNA FFPE Kit. For each sample, 100,000 sequencing reads were subsampled from alignment files and the number of non-polymorphic, high-quality mismatches (base quality≥30) per megabase was calculated. The Wilcoxon signed-rank test was used to compare the mismatch rates between DNA isolated from the two kits. The same method was used to compare the mismatch rates in DNA isolated from the QIAamp DNA FFPE Tissue Kit alone and those in DNA that was further processed with the NEBNext FFPE DNA Repair v2 protocol.

### Mutational signature analysis

In samples with more than 100 mutations, SomaticSignatures (v2.24) was used to extract mutational signatures. Signatures were compared to known COSMIC mutational signatures (v3) (Alexandrov et al., 2020) using cosine similarity.

### SCNAs

CopywriteR (Kuilman et al., 2015), which uses off-target sequencing reads to generate DNA copy number profiles, was adapted for use with canine and feline sequencing data. The modified code is available on GitHub at https://github.com/OscarKrijgsman/CopywriteR_dogs_cats. DNA copy number profiles were segmented using CBS (Venkatraman and Olshen, 2007), after which the profiles were normalized based on the mode of the segmented values. PurBayes (Larson and Fridley, 2013) was used to estimate tumor sample purity, and log2 ratio cutoffs were adjusted accordingly to call regions of somatic copy number gain or loss. For replicate tumor samples, the sample with the highest purity estimate was used for downstream analyses.

### Somatic mutations in human AS

For comparison of canine and feline HSA to human AS, data from 62 patients from the Angiosarcoma Project (Painter et al., 2020) were downloaded from the project's cBioPortal web page (Provisional, July 2020). For patients with data from samples with different time points, the sample from the earliest time point was used. Blood biopsies were excluded. The samples are listed in Table S5 and data used here can be accessed from our virtual study: https://www.cbioportal.org/study/summary?id=5ff4d5cae4b015b63e9d324f.

### Germline variants in orthologs of human HSA risk genes

From the germline variant calls generated from Pisces (described above), we searched for potential variants of interest in canine and feline orthologues of human AS risk genes *TP53*, *PIK3CA*, *POT1*, *PTEN*, *RB1*, *IDH1*, *IDH2*, *XPC* and *ERCC2* (Cao et al., 2019). *PTHR1* and *POLH* are also AS risk genes; however, these genes were not included in our cancer gene panel. To remove false variants in the canine germlines that were due to reference genome errors, the LiftOver tool (Kuhn et al., 2013) was used to check the equivalent genomic position in the reference genome CanFam4.

### Analysis of canine *TP53* RNA-seq reads

RNA-seq data from canine HSA (Megquier et al., 2019) were downloaded from the NCBI Sequence Read Archive BioProject PRJNA562916 (BioSamples SAMN12659339-SAMN12659361) and aligned to the CanFam3.1 reference genome using the STAR (v2.5.0c) aligner (Dobin et al., 2013). The STAR genome index files were created using gene models from Ensembl v98. Alignments to *TP53* were visualized using the Integrative Genome Viewer (Robinson et al., 2011).

## Supplementary Material

Supplementary information
