# Supplementary material for: Comparison of the oncogenomic landscape of canine and feline hemangiosarcoma shows novel parallels with human angiosarcoma
Source: Dis Model Mech. 2021 Jul 23;14(7):dmm049044. doi: 10.1242/dmm.049044 (PMC8319545; doi:10.1242/dmm.049044)
Supplement: Supplementary information [file dmm-14-049044-s1.pdf]

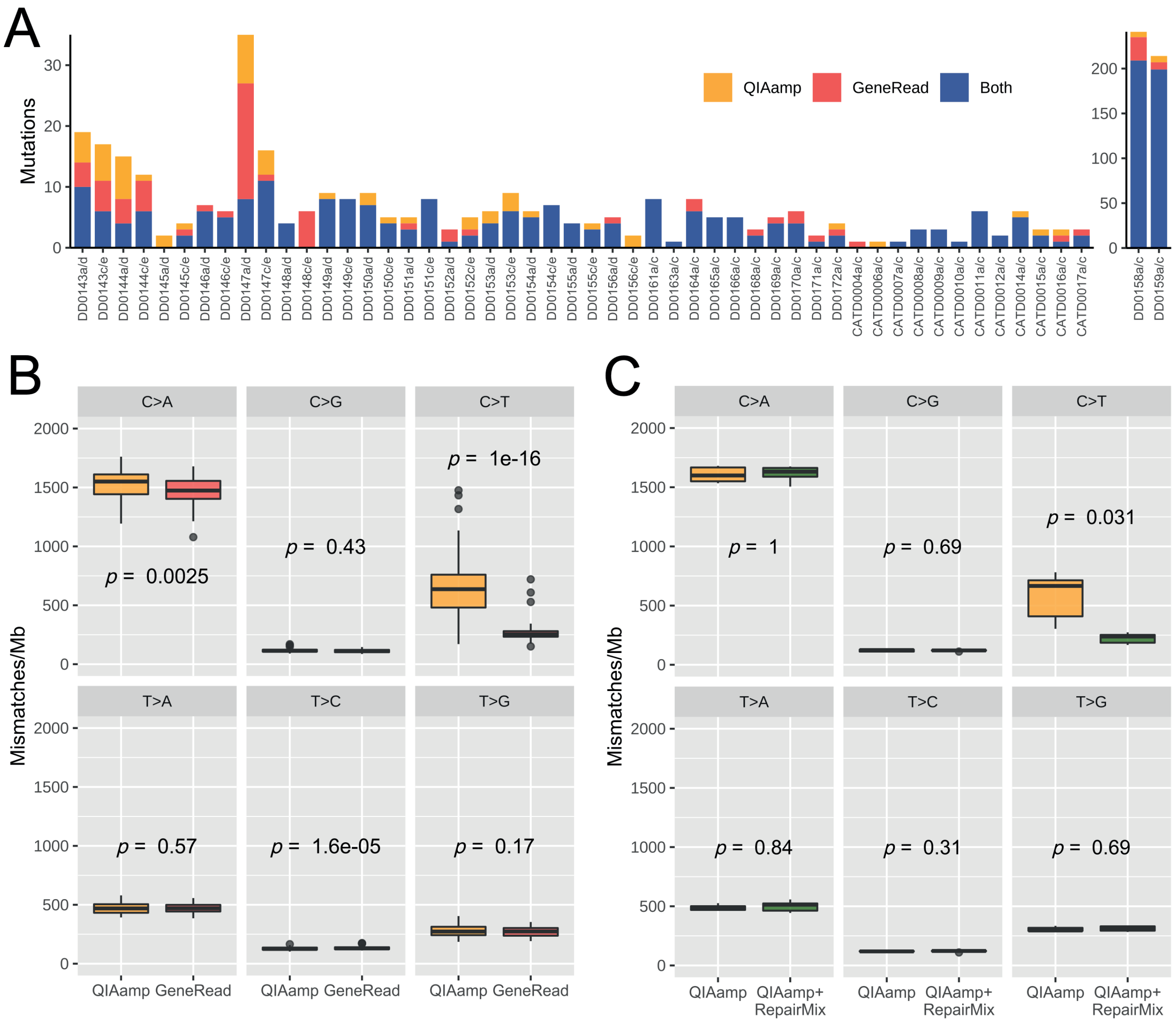

Disease Models & Mechanisms • Supplementary information

Fig. S1. Comparison of FFPE tissue DNA extraction kits. (a) Somatic single and dinucleotide variant calls shared and unique to biological replicate samples processed with the QIAamp DNA FFPE Tissue Kit and the GeneRead DNA FFPE Kit. The variants shown were compared after filtering for C>A/G>T and C>T/G>A artefacts, but prior to comparison to Pisces (see **Materials and Methods**). In canine and felines samples suffixed with 'a/c', 'a' samples were processed with the QIAamp DNA FFPE Tissue Kit. For canine samples suffixed with 'a/d' and 'c/e', the 'a' and 'c' samples were processed with the QIAamp DNA FFPE Tissue Kit. Samples from DD0158 and DD0159 are samples that had a UV mutational signature. (b) Comparison of the mismatch rate in sequencing reads generated from DNA isolated from the Qiagen and GeneRead kits. High-quality mismatches relative to the reference genome were used to estimate the level of artefacts (see **Materials and Methods**). Both tumor and germline samples are included (n=92 for each kit). As expected, C>T/G>A were significantly reduced in the GeneRead kit. (c) Six samples (3 tumors and 3 matched germline samples) were processed with the QIAamp Kit; half of the DNA from each sample was subjected to a DNA repair step (see **Materials and Methods**). The C>T/G>A artefacts are significantly reduced in the sample subjected to the DNA repair step. Statistical significance was calculated using the Wilcoxon signed-rank test.

Disease Models & Mechanisms • Supplementary information

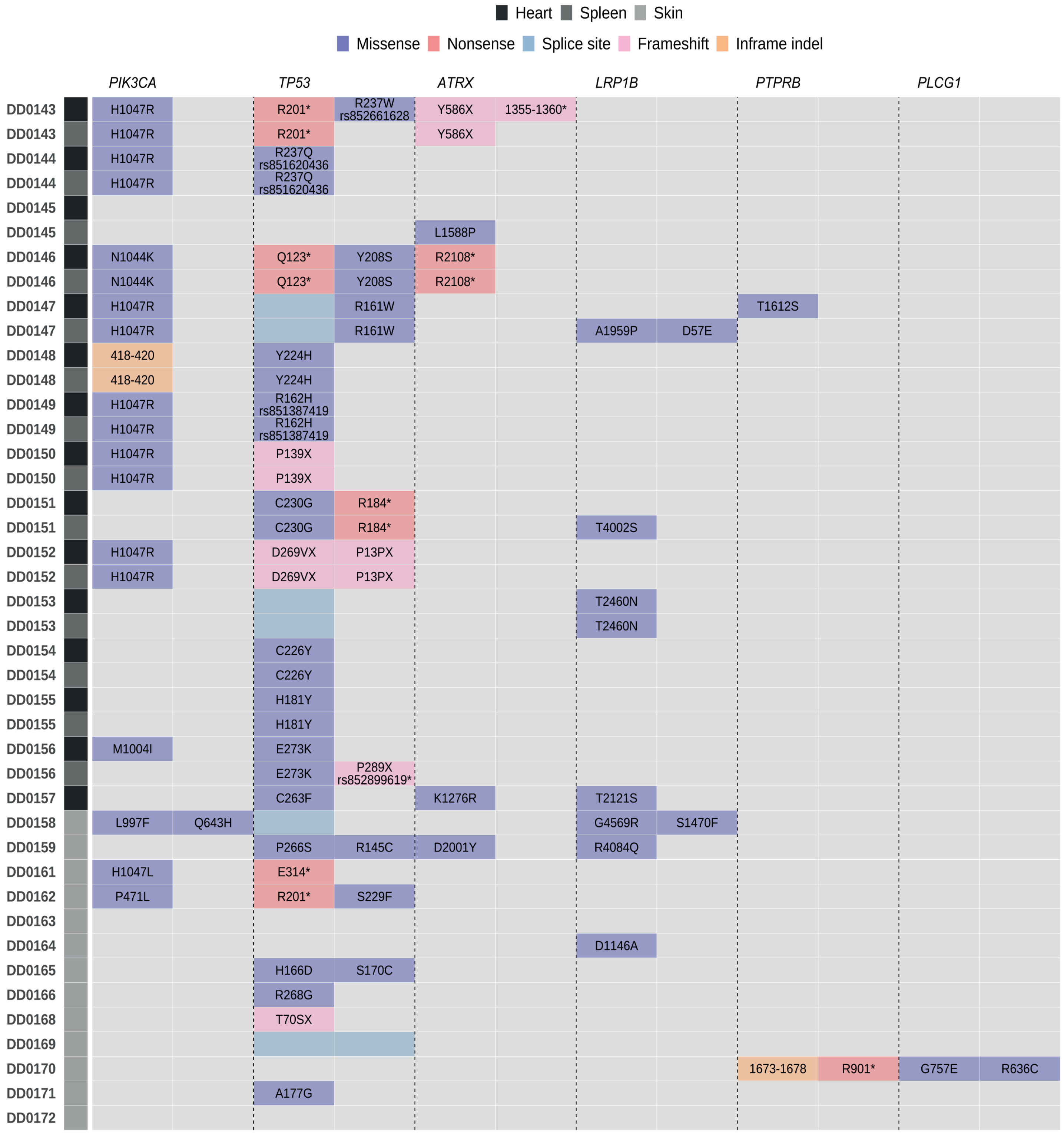

Fig. S2. Genes with multiple mutations. Included here are genes with more than 1 mutation in at least one tumor. Also shown are the corresponding protein changes for each mutation. An rsID is provided if the exact variant was present in dbSNP (v150); an rsID with an asterisk indicates a variant found in the same position with a different variant allele. The ATRX frameshift deletion at amino acid position 1355-1360 in DD0143 is linked to a missense mutation at position amino acid position 1348. In almost all cases, mutations are shared between tumors from the heart and spleen from the same dog. There are no genes with multiple mutations in any individual feline tumor.

Disease Models & Mechanisms • Supplementary information

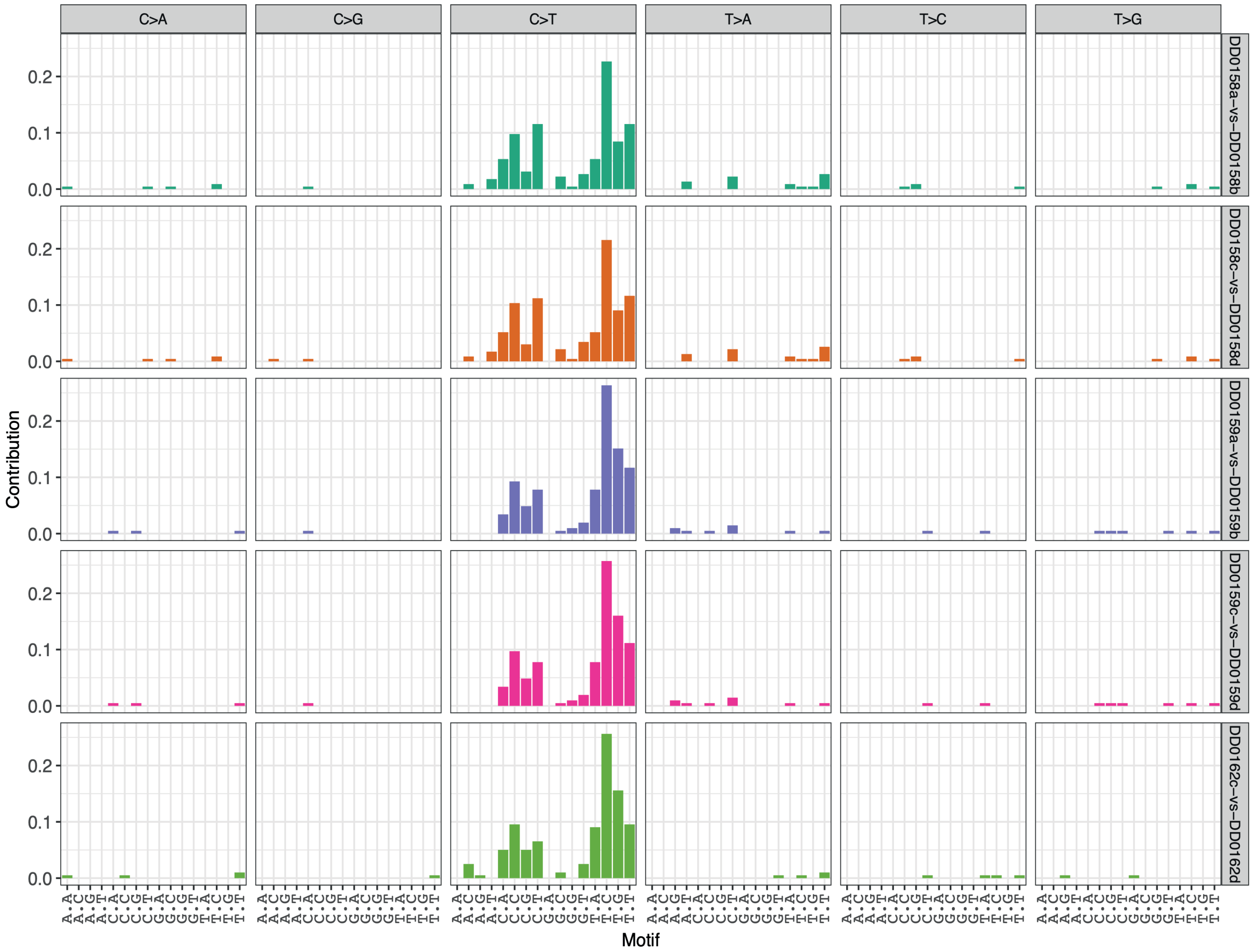

Fig. S3. Mutational spectra of canine HSA samples with a UV mutational signature. Shown are the spectra from five cutaneous HSA samples from 3 canine patients. Replicate samples are shown for patients DD0158 and DD0159. Contribution is the proportion of total mutations. COSMIC mutational signatures SBS7a and SBS7b, which are associated with UV light exposure, were identified in these samples.

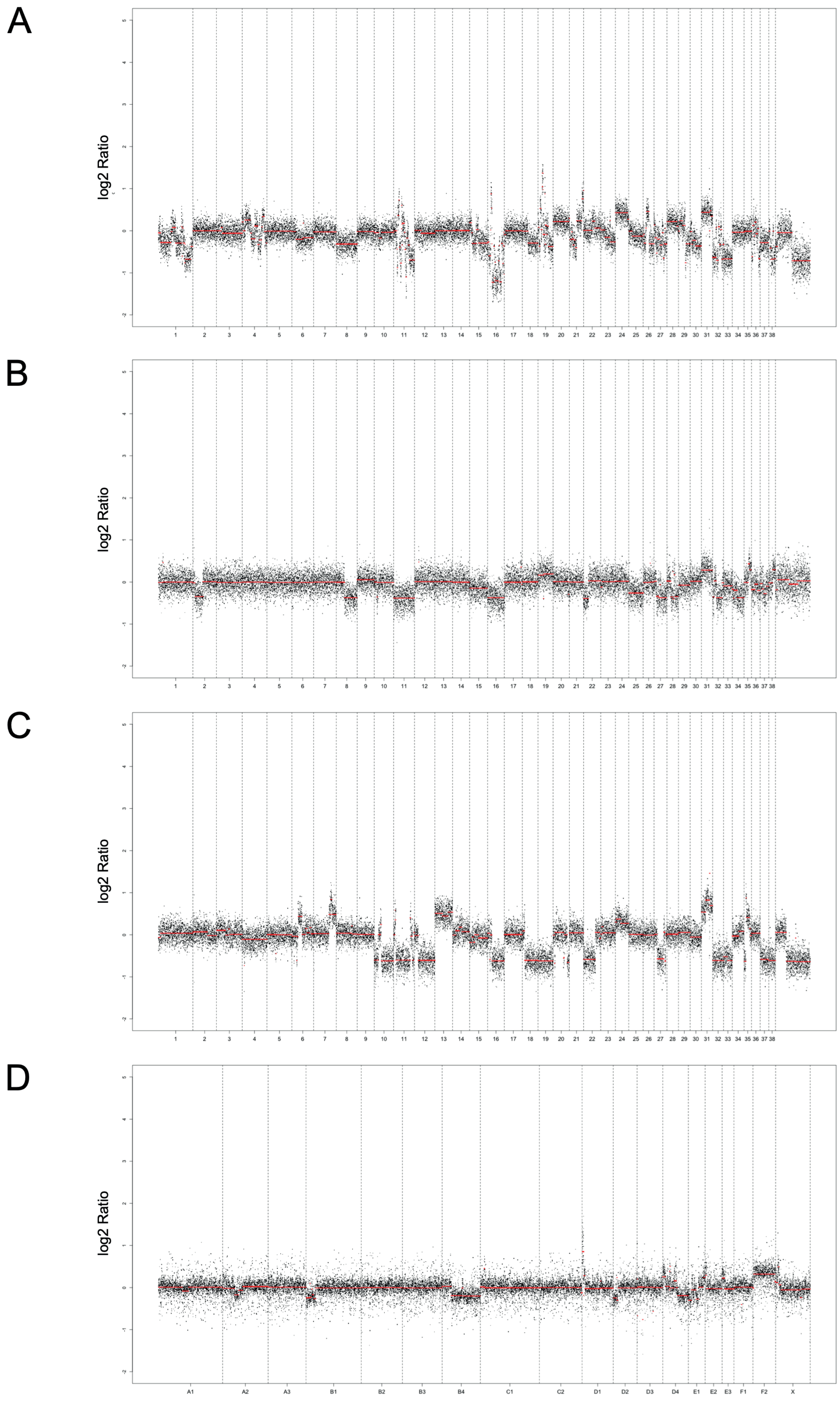

Disease Models & Mechanisms • Supplementary information

Fig. S4. Examples of DNA copy number profiles produced by CopywriteR. Shown are bin-level log2 ratios and segmentation calls (red) for (a) canine HSA samples DD0150d, DD0152e, DD0168c and (b) feline sample CATD0013c.

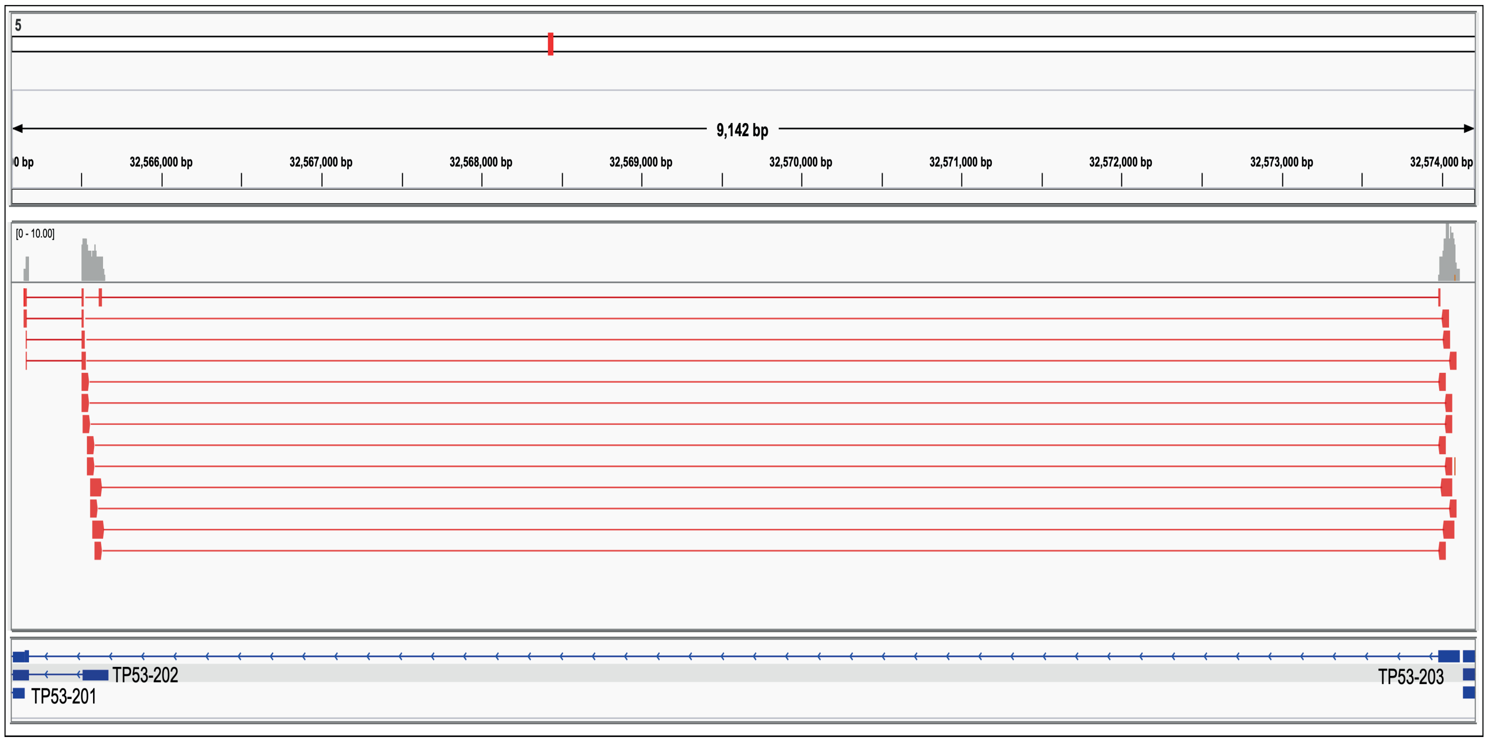

Fig. S5. Alignment of RNA-seq reads to exon 1 of canine TP53-202. Represented here are the 5’ ends of the three TP53 transcripts (blue) in Ensembl v98, relative to the reference genome CanFam3.1. *TP53* is on the reverse strand. Alignment of RNA-sequencing reads from canine HSA (Megquier et al., 2019) provides evidence that there is an exon upstream of the predicted TP53-202 transcript and therefore the Ensembl v98 TP53-202 transcript annotation is erroneous.

Table S1. Clinical details of the patients in the study. Sample and patient information for (a) canine and (b) feline samples included in this study. Samples from the same patient and tissue were processed with 2 different DNA extraction kits. DSL is domestic shorthair; DLH is domestic longhair. Sex is male (M), female (F), or unknown (U). Age is age at diagnosis; U, unknown age.

Click here to download Table S1

Table S2. Canine and feline orthologs of human cancer genes included in the target enrichment bait sets. (a) Canine genes and (b) feline genes included in bait sets, and human orthologs. Ensembl v98 was used to find orthologs and orthology relationships. For human genes with no canine/feline ortholog, canine and feline genes were selected based on gene symbol. For canine and feline genes not assigned a gene symbol, only the ENSEMBL gene ID is listed.

Click here to download Table S2

Table S3. Protein-altering variants in feline and canine HSA. Protein-altering variants in (a) canine and (b) feline HSA, including missense, nonsense, splice site, frameshift and inframe indel mutations. VEP consequence is the predicted variant effect from the Ensembl Variant Effect Predictor. Tumor_ID/Normal_ID indicate the matched tumor/normal samples; Patient_ID indicates the individual dog or cat. Note that an individual dog or cat may have more than 1 sample, from the same tumor and/or different sites (heart/spleen). VAF is the variant allele frequency in the tumor sample. Alt_Allele_Forward/Reverse is the number of reads supporting the alternate allele, mapped on the forward and reverse strand. Variant_ID indicates if the variant was found in dbSNP, the Dog Genome Project (DGP), or Dog Genome SNP Database (DoGSD v2). Sex is male (M), female (F), or unknown (U). For canine and feline genes not assigned a gene symbol, only the ENSEMBL gene ID is listed.

Click here to download Table S3

Table S4. Germline variants in orthologs of human angiosarcoma risk genes. Protein-altering variants in the germlines of (a) canine and (b) feline patients with HSA. VEP consequence is the predicted variant effect from Ensembl Variant Effect Predictor. Patient_ID represents the individual dog or cat. If more than 1 germline sample was available, a variant is shown if it was present in at least 1 germline sample. Variant_ID indicates if the variant was found in dbSNP, the Dog Genome Project (DGP), Dog Genome SNP Database (DoGSD v2), or Cat Genome Sequencing Initiative (CGSI). Canonical indicates whether the annotated conseqeunce is relative the the canonical transcript as defined in Ensembl v98. Sex is male (M), female (F), or unknown (U).

Click here to download Table S4

Table S5: Samples from the Angiosarcoma Project used for comparison with canine and feline HSA. Sixty-two samples from 62 patients in the Angiosarcoma Project cohort were selected (cBioPortal, July 2020). For each patient, the samples from the earliest time point was selected, excluding blood biopsies.

Click here to download Table S5
